# Management of recurrent hip dislocation in Down Syndrome using modified Ganz periacetabular osteotomy: Follow up after 5 years

**DOI:** 10.1016/j.amsu.2020.04.021

**Published:** 2020-05-03

**Authors:** Ismail Hadisoebroto Dilogo, Jessica Fiolin, Juniarto Jaya Pangestu, Amri Muhyi

**Affiliations:** Department of Orthopaedics and Traumatology, Faculty of Medicine Universitas Indonesia, Dr. Cipto Mangunkusumo General National Hospital, Jl. Diponegoro No. 71, Jakarta Pusat, Indonesia

**Keywords:** Recurrent posterior hip dislocation, Down syndrome, Dysplastic hip, Modified Ganz periacetabular osteotomy

## Abstract

Recurrent hip dislocation in a Down Syndrome patient with dysplastic hip is a very rare and challenging case to treat even for an expert orthopaedic hip surgeon. Least compliant patient and family, lowly educated with low socioeconomic status, young age and several anatomical variations forces limited option as a treatment. Several literatures mentioned Despite requiring only minimal implant, this technically demanding surgery requires a thorough understanding of the hip anatomy. This is the first case worldwide reporting 5 year follow up of dysplastic hip with DS treated successfully with periacetabular osteotomy (PAO) technique. An eighteen years old female with DS had multiple posterior hip dislocation episodes since 3 years prior our hospital admission. A modified Ganz PAO was performed under image intensifier guide. Patient was able to talk and hip was never dislocated again within 5 years follow up. Ganz periacetabular osteotomy, although a technically demanding surgery, is a preferable treatment in recurrent hip dislocation for Down Syndrome patient with good to excellent clinical and radiological outcome.

## Highlights

• Recurrent hip dislocation in DS is a very rare and challenging case.
• The first case worldwide reporting 5 year follow up of dysplastic hip with DS.
• Within five years after surgery, the hip have never been dislocated again.

## 1 Introduction

Recurrent hip dislocation in a Down Syndrome (DS) patient with dysplastic hip is a very rare and challenging case to treat even for an expert orthopaedic hip surgeon. Least compliant patient and family, lowly educated with low socioeconomic status, young age and several anatomical variations forces limited option as a treatment. This is a case of the very rare success report of recurrent hip dislocation alternative treatment, the latest was 20 years prior by Gore, 1999 [1]. This technically demanding surgery requires a thorough understanding of the hip anatomy. This is the first case worldwide reporting 5 year follow up of dysplastic hip with DS treated successfully with periacetabular osteotomy (PAO) technique. This paper was written according to the SCARE guideline [2].

## 2 Presentation of case

An eighteen years old female with DS had multiple posterior hip dislocation episodes since 3 years prior admission to the central referral hospital in Indonesia. Previously patient was able to walk though her previous gait was unknown. Patient's caregiver admitted the first event was due to fall from stairs and was brought to a nearby bonesetter and performed a traction. Ever since then, patient could not walk and even repeatedly fall with recurrent hip dislocation and taken to bonesetter due to economic problem. However during the last event the bonesetter could not reduce and patient was referred to our center.

Patient's parents were divorced rendering her grandmother to carry out as a caregiver. A general investigation of patient was carried out together with pediatric department showing no relevant history in the family and despite patient's distinguished clinical feature (flat face, almond eyes, short neck, small ears, palmar crease, and poor muscle tone) there have been no other internal organs anomaly hence patient was otherwise healthy and did not take any drugs.

**Fig. 1:**
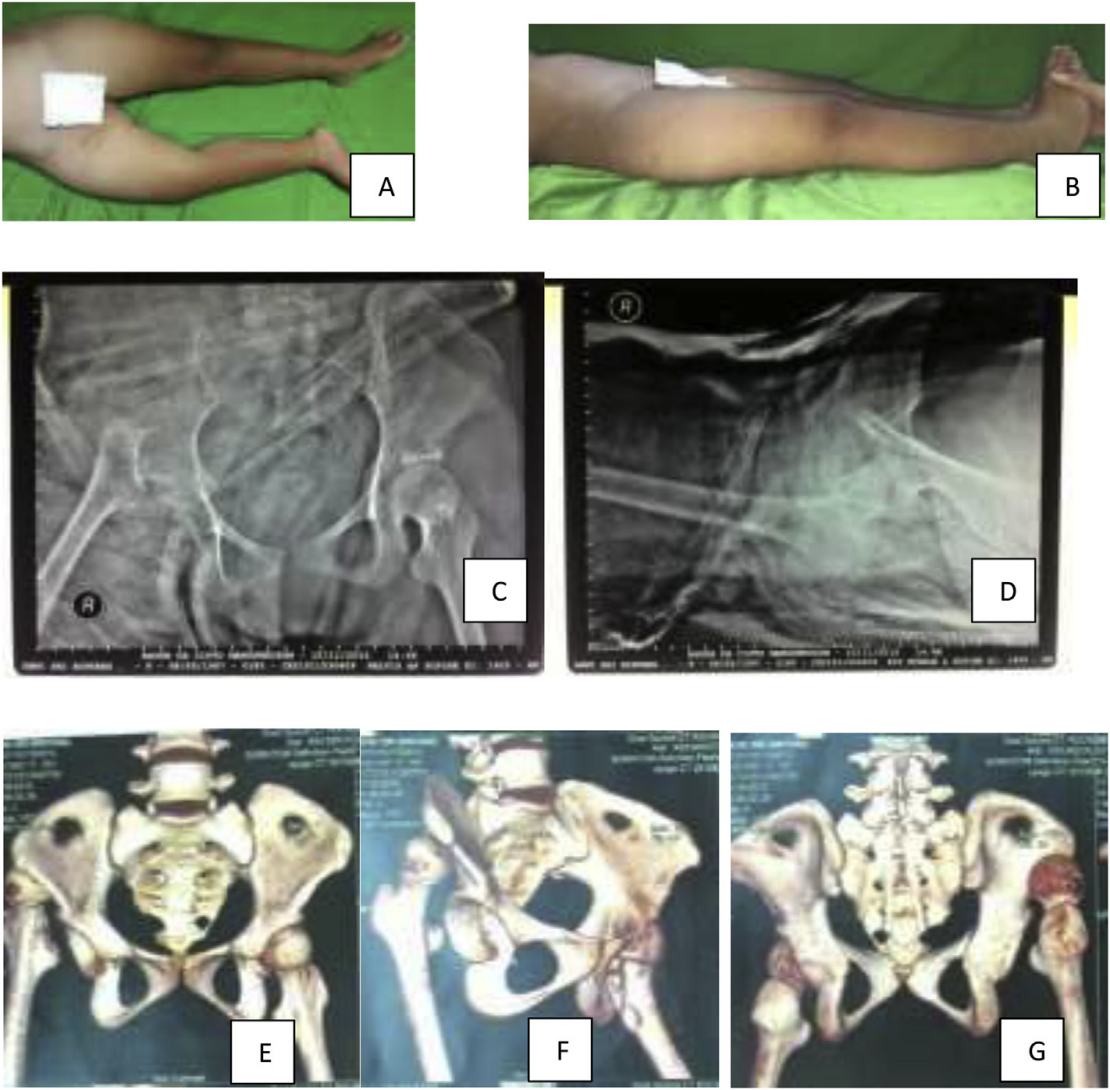
A-B showed clinical examination of patient. Female, a 5 cm leg length discrepancy was noted. C-D showed Pelvic AP and axial Xray. Radiograph of failed closed reduction and spica application showing a persistent dislocation. Acetabular index of right hip were 55^0^ and epiphyseal plate has closed. E-G showed Pelvic CT 3D reconstruction. Right posterior hip dislocation, with no concomittant fracture in acetabulum nor femoral head, shape of femoral head was still round.

The right hip was flexed and externally rotated, there was a 5 cm of leg length discrepancy and neurovascular was intact (Fig. 1).

Several attempts of close reduction and hip spica applications under sedation with guided image intensifier were performed in the emergency room by chiefs orthopaedic resident on duty upon dislocation despite no successful retaining of reduction (Fig. 1). A computed tomography (CT) scan was ordered showing a posterior hip dislocation with shallow acetabulum and no associated femoral head and acetabular fracture. We also found that the shape of femoral head was still round despite multiple episodes of dislocation (Fig. 1).

**Fig. 2:**
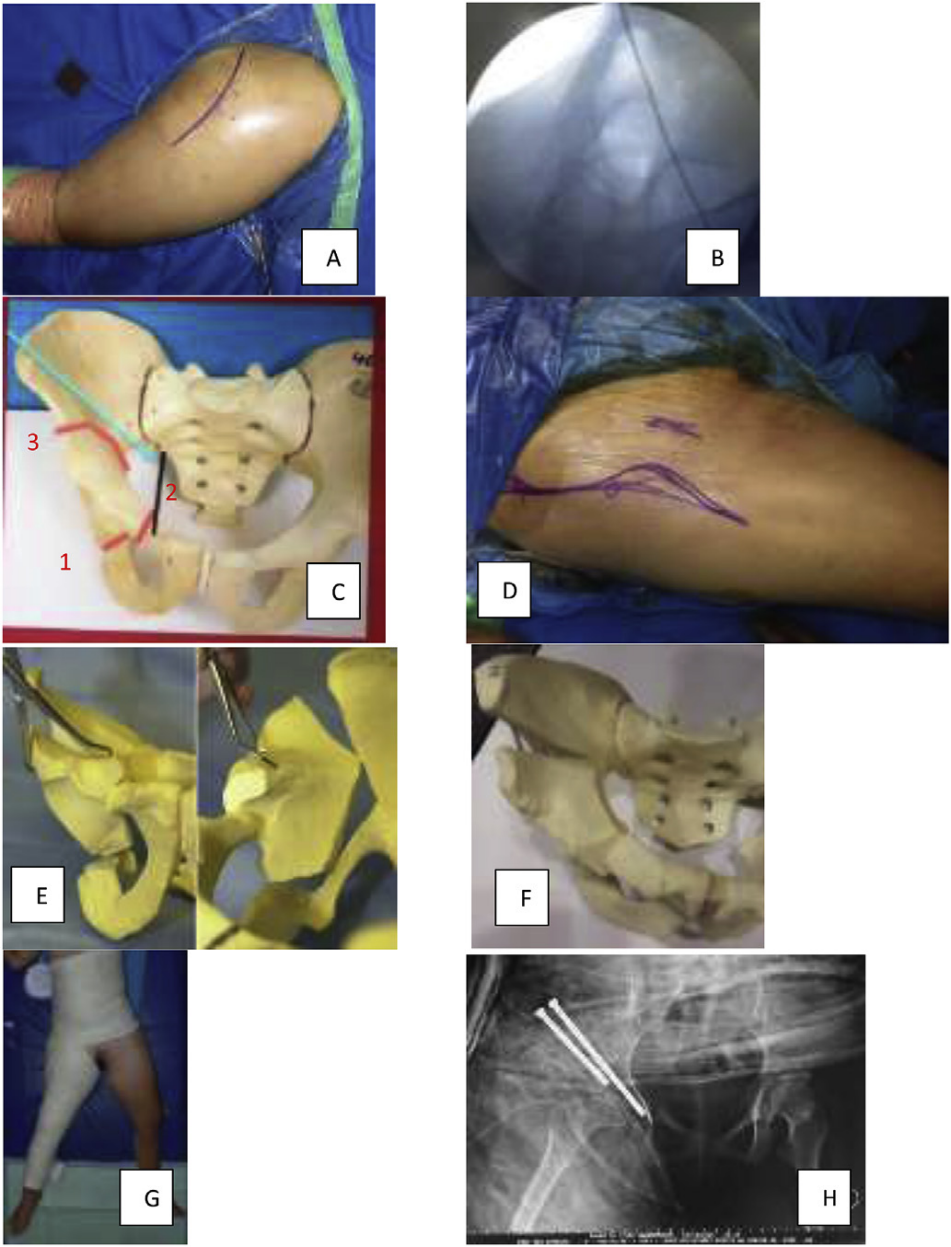
Surgical technique of modified PAO. A, patient was positioned in lateral decubitus; B, hip was reduced and confirmed using image intensifier (II); C1-3, osteotomy of ischium was performed first then followed with superior ramus pubis and acetabulum using anterior approach; D, patient then positioned supine and anterior approach were performed to proceed with C2-3; E, derotation was performed; F, fixation using cannulated screw and bone graft application; G, post-operative hemi spica cast was applied and; H, post-operative xray showing increased well contained hip.

Patient and caregiver were consented and prepared for an open reduction and reconstruction surgery using PAO technique by adult reconstruction consultant and team. Patient was positioned in lateral decubitus initially, then the hip was exposed and reduced using Southern-Moore posterior approach. After open reduction, osteotomies were performed to maintain hip containment in the ischium, then continued with capsulorrhaphy (Fig. 2). After the wound was closed, patient was turned supine and the hip was exposed using Smith-Peterson anterior approach and osteotomy of superior ramus pubis and iliac bone were performed (Fig. 2). A block of tricortical graft from iliac crest was harvested to fill in gaps. Then, derotation maneuver was performed under image intensifier to obtain adequate coverage followed with bone graft and fixation using 2 cannulated screw and hip spica cast application (Fig. 2).

Within five years after surgery, the hip have never been dislocated again, patient could sit without pain and walk with full weight bearing with aid gradually although Harris Hip Score could not be performed due to Down Syndrome. Patient's caregiver was very satisfied with the procedure and stated that patient which was previously often upset do to pain and discomfort, was never upset again 1 month after the surgery, after the wound had been healed and hip spica was removed due to the inability of patient to comply for instructions.

**Fig. 3:**
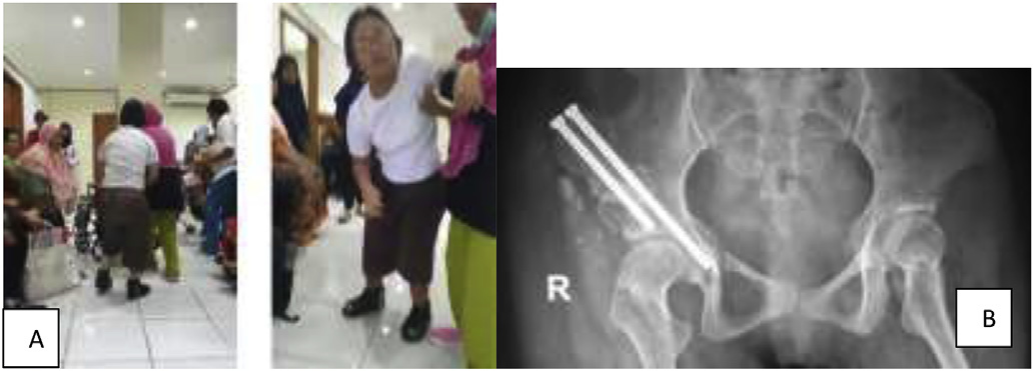
Post-operative clinical examination and radiograph after 5 years follow up. A, clinical ability to stand full weight bear with aid; B, 5 years post-op radiograph showing union at osteotomy sites, and concentric hip.

Within 3 months patient started to stand with aid and walk started at 12 month. Normal range of motion of the hip was observed after the 4th month with 120^0^ flexion, 40^0^ abduction, 30^0^ internal rotation, and 45^0^ of external rotation. Leg length discrepancy was negligible (0.5 cm), fracture has fully united within 6 months and acetabular index was 30^0^ (Fig. 3).

## 3 Discussions

Patients with DS naturally possess the characteristics of ligamentous laxity, muscle hypotonia and gait abnormalities [3,4]. Hip dysplasia and a number of acetabular, femoral or combined femoro-acetabular anatomical variations are not uncommon. Several previous studies reported the use of arthroplasty as a treatment to the unstable hip in DS cases [5]. There are many drawbacks in using arthroplasty of patients with Down syndrome, i.e: dysplastic acetabulum, different neck-shaft angle, very narrow femoral canal and posteriorly oriented greater trochanter, hence the need to prepare a more specific and higher cost implants which are not readily available in our center, and lastly the longevity of implant [6,7]. As our patient was still young and the life expectancy of these patients continues to increase, we fought to find an alternative treatment which could delay the need for arthroplasty.

Another literatures mentioned the femoral varus derotation osteotomy also useful to reduce the dislocated femoral head [[8], [9], [10]].

A periacetabular osteotomy performed originally by Ganz was aimed to increase the life of hip joint by restoring the proper shape of the hip joint [11]. Originally, Ganz was performed through an anterior only approach however due to the lack of instruments we modified using the double approach; first from prone position and posterior approach to cut the ischium bone, and then proceed to supine and anterior approach to osteotomy ilium and pubic bone. Afterward, the whole structure that formed the acetabulum was reshaped into a more anteverted and deep acetabular roof then fix it using the cannulated screw under image intensifier. Soft tissue correction by capsullorraphy was also performed to maintain the contained hip. Hip spica cast is a must since the patient could not follow instructions due to the Down Syndrome.

Patient's healing rate was indifferent with normal patient as in wound healing and bony union period. However, functional ability as walking was lower than normal (12 months as opposed to 6 months) due to poor muscle tone and balance coordination inherited in a DS patient.

This procedure also proves its longevity with such inexpensive implants. At five-year-annual follow up, patient and her caregiver did not have any complain nor pain. Range of motion was nearly normal, patient could sit and walk without pain. Radiographic x-ray also shown good containment with all osteotomy cuts have united. However, the very rare number of case and a good knowledge of anatomy rendering this technique unpopular. Therefore, larger studies with longer follow up time to evaluate the needs of arthroplasty conversion should be performed.

## 4 Conclusions

Ganz periacetabular osteotomy, although a technically demanding surgery, is a preferable treatment in recurrent hip dislocation for Down Syndrome patient with good to excellent clinical and radiological outcome.

## Consent of patient

Written informed consent was obtained from the patient for publication of this case report and accompanying images. A copy of the written consent is available for review by the Editor-in-Chief of this journal on request.

## Sources of funding

None.

## Author contribution

Ismail Hadisoebroto Dilogo (1st author): Conceptualization, Investigation, Writing – Review & Editing, Supervision.

Jessica Fiolin (2nd author): Resources, Writing – Original Draft, Visualization, Project Administration.

Juniarto Jaya Pangestu (3rd author): Resources, Writing – Original Draft & Editing.

Amri Muhyi (4th author): Resources, Writing – Original Draft & Editing (Annals of Medicine and Surgery format).

## Registration of Research Studies

Our study is a case report study

## Guarantor

The guarantor is the first author, Ismail Hadisoebroto Dilogo, who accept full responsibility for all decision about this paper

## Consent

Written informed consent was obtained from the patient for publication of this case report and accompanying images. A copy of the written consent is available for review by the Editor-in-Chief of this journal on request.

## Provenance and peer review

Not commissioned, externally peer reviewed.

## Declaration of competing interest

None declared.
